# New Appliances and Things Medical

**Published:** 1896-08-01

**Authors:** 


### NEW APPLIANCES AND THINGS MEDICAL.

[We shall be glad to receive, at our Office, 428, Strand, London, W.C., from the manufacturers, specimens of all new preparations and appliances, which may be brought out from time to time.]

### ROSBACH SPRINGS (LIMITED).

(38, Leadenhall Street, London, E.C.)

Of all the table waters in the market we consider Rosbach to be on the whole the purest, the most palatable, and the best. Bright, pleasant to the taste, and full of sparkle, it deserves to be better known than it is, as it is free from certain objections which attach to some other table waters in the market. The water is bottled at the springs near Homburg, and is supplied in excellent condition for use. We have used Rosbach for years, because we have found it to be the pleasantest and most palatable of table waters.

### MINIATURE AMBULANCE CASE.

(Messrs. Maw, Son, and Thompson, 7, Aldersgate Street, E.C.)

A very useful little contrivance for use in emergencies has been brought out by Messrs. Maw. It consists of a small tin case containing boracic lint, adhesive plaster, ammonia, silk, two bandages, compress, safety, and surgical pins, needle, and tapes. The whole only occupies a few inches space, and can be easily carried in the pocket or in the bicycle satchel. Simple directions are given as to the use of the contents. It would form a useful little present to any bicyclist, for travellers, or others likely to incur accidents at a distance from skilled help.

